# Influence of soft coral density and depth on benthic communities on Fiji's Rainbow Reef

**DOI:** 10.1098/rsos.260513

**Published:** 2026-11-04

**Authors:** Nile P. Stephenson, Katie M. Delahooke, Charlotte G. Kenchington, Tasnuva Ming Khan, Lea Katz, Jone Waitaiti, Alice A. Ball, Victor Bonito, Emily G. Mitchell, Andrea Manica

**Affiliations:** ^1^ Department of Zoology, University of Cambridge, Cambridge, UK; ^2^ Department of Earth Sciences, University of Cambridge, Cambridge, UK; ^3^ University Museum of Zoology, University of Cambridge, Cambridge, UK; ^4^ King's College, University of Cambridge, Cambridge, UK; ^5^ British Antarctic Survey, Cambridge, UK; ^6^ Marine Biology Lab, Université Libre de Bruxelles, Brussels, Belgium; ^7^ Viani Bay Dive School, Vanua Levu, Fiji; ^8^ Reef Explorer Fiji, Votua Village, Fiji

**Keywords:** soft coral, spatial ecology, Bayesian network inference, Fiji, facilitation

## Abstract

Some soft corals have the potential to increase in prevalence on tropical coral reefs as the severity of anthropogenic climate change increases. While soft corals are therefore an increasingly important component of reef ecosystems, little is known about their ecological role on coral reefs and their influence on community dynamics and diversity. We used Bayesian network inference (BNI) to identify the associations among benthic taxa across sites with varying degrees of soft coral dominance on Fiji's Rainbow Reef, and then employed spatial point process analysis (SPPA) to reveal the ecological processes behind these associations. We found that depth was a dominant driver of community dynamics, and that white Nephtheidae soft corals were negatively associated with scleractinian corals and positively associated with algal proliferation—possibly owing to soft corals reducing grazing pressure. Our results show distinctions in reef benthos and ecological dynamics between scleractinian and soft coral-dominated components of the Rainbow Reef. We found that diversity levels were significantly lower on soft coral-dominated quadrats relative to scleractinian-dominated quadrats, potentially highlighting the reduction in benthic diversity when Nephtheidae soft corals dominate reef walls.

## 1. Introduction

Tropical coral reefs are among the most biodiverse ecosystems on Earth [1] but are under threat from anthropogenic climate change [2–5]. As the frequency and severity of threats such as marine heatwaves, ocean acidification, pollution and overharvesting increases, coral reef communities are expected to change in species composition [5–7], with an increase of organisms that are more resistant to the effects of climate change [8–11]. Coral reefs are dominated by scleractinian corals, but soft corals are a common occurrence [12,13] and sometimes form carpets of local dominance [14,15]. While some soft corals are susceptible to the same pressures as scleractinian corals [11,16,17], many are resistant to, or benefit from, changing climate conditions because of physiological adaptations (e.g. nitrate eutrophication in *Xenia*, tolerance of acidification in *Sarcophyton* and *Veretillum*) or warming-resistant symbiont host dynamics [6,7,11,18,19]. As such, it is possible that soft corals will play a more dominant role in coral reefs under future climate scenarios [6,20–22].

The biodiversity benefits of scleractinian coral reefs are emergent properties of both the composition and structure of reefs [23,24], and their ecological dynamics [25]. It is unclear how ecological dynamics will change if soft corals become more prevalent [20] because much of the biodiversity generated by scleractinians is attributable to ecosystem engineering [24,26]. Many soft corals do not produce such benefits because they provide only temporary habitat owing to periodic retraction of their bodies and only over their own lifetimes [27]. It is therefore important to understand the ecological dynamics of soft coral reefs to understand how reefs may change as soft corals increase in prevalence, and how these changes will affect reef biodiversity.

Ecological associations within communities can be identified via Bayesian network inference (BNI), which elucidates conditional dependencies between variables (in an ecological context: species or environmental characteristics) [28–31]. In BNI, nodes (in an ecological context: species, environment) are connected by edges (inferred ecological associations). Edges have a sign (positive, negative) denoting the nature of the interaction and the influence score (IS) denotes the interaction's strength [32]. Bayesian networks (BNs) are scale-agnostic, so variables can include biotic (species, genes) at a variety of taxonomic levels and abiotic (space, time, depth) variables [29,30,32,33]. Associations identified by BNs are reflections of conditional correlations across variables, which may reflect a wide range (and potentially a combination) of ecological dynamics (e.g. competition, habitat associations, trophic interactions).

The scope of ecological techniques such as BNI to capture specific dynamics is dependent on the data available [28]. Few studies, if any, can measure every aspect of a community—many studies will focus exclusively on, for example, the benthos or the nekton, but not both, even though there are clear interactions between the two (e.g. [34]). Different ecological associations yield diverse patterns in abundance distributions—some processes can generate very strong patterns (e.g. distinct habitat preferences, competitive exclusion [35]). Some ecological processes may generate more nebulous patterns, because these processes depend on a wide variety of ecological contexts (e.g. trophic interactions, intransitive competition [36]). Furthermore, ecological dynamics that have been generated by unmeasured variables may present themselves in BNs as ‘direct’ interactions (e.g. an edge between two nodes, A → C), but may actually reflect the effects of (a set of) unmeasured ecological variables [37]. Identifying direct interactions as true positives versus false positives (A → B → C, where B has not been measured but is the true agent of the observed association between variables A and C) is impossible without direct observation or prior knowledge of a system [28,30]. As such, patterns identified in BNs (and, indeed, other similar ecological approaches, e.g. [37]) represent associations that are present in the data but which do not necessarily represent true direct interactions [38]. The combination of indirect effects and patterns that can be generated by a range of different ecological processes can therefore make interpretation of these approaches challenging. BNs offer an advantage over other methods in that they can establish conditional relationships which are often present in natural systems [28–30,39] should suitable data be available. In addition, BNs, like other approaches in community ecology, present a method to identify associations that can, in turn, be secondarily tested using a suit of other analyses (or, potentially, with other data) [40].

Even when true positive ecological associations are found by BNs, ecological drivers cannot be determined by BNs alone [31,33] because correlations between variables may reflect a range of ecological processes occurring over an organism's life history. Pairwise ecological processes that drive associations within sessile species can be inferred using spatial point process analysis (SPPA) because the spatial patterns of sessile organisms reflect the possible underlying processes that have occurred throughout an organism's life [41–43]. Points (organism locations) of a pair of species can be: (i) random, reflecting no associations; (ii) aggregated, reflecting aggregation of points between populations (denoting, e.g. mutualisms, shared habitat associations); or (iii) segregated, reflecting hyperdispersion of points of each population (denoting, e.g. mortality incurred by competition) [43,44]. Each of these patterns (random, aggregation, segregation) can occur across multiple spatial scales and at different strengths. For instance, segregation at small scales and aggregation at large scales may indicate the influence of resource competition at small scales and shared habitat preferences at larger scales [44]. The most likely underlying processes can be assessed by model-fitting of patterns of known processes enabling inference of those underlying processes [43,45].

In this study, we applied BNI and SPPA sequentially to the benthos of three sites on the Rainbow Reef, Fiji to assess how ecological dynamics may vary with soft coral dominance. BNI was performed on all sites together to elucidate the possible ecological associations on the Rainbow Reef, and subsequent SPPA was used to investigate the dynamics underpinning these associations. We subsequently used linear models to understand how changing soft coral dominance influenced benthic biodiversity metrics.

## 2. Material and methods

### 2.1. Field site

In this study, we focused on three sites on the Rainbow Reef, in the Somosomo Strait, Fiji. The Great White Wall (GWW) is an ideal site to study soft coral ecological dynamics because it has a large spatial extent of the vertical wall dominated by a carpet of white Nephtheidae [46]. To understand how ecological dynamics differed between sites of variable soft coral dominance, we added two more sites: an adjacent reef wall (ARW), a nearby (within approx. 100 m) reef wall but with fewer white Nephtheidae, more scleractinians and a mixed filter-feeding community; and the upper forereef (UFR) area, a shallower, more horizontally oriented habitat sitting just above the GWW and ARW, dominated by scleractinians and colourful *Dendronephthya* soft corals. These three closely connected sites represent the three core habitat types we observed on this part of the Rainbow Reef (figure 1).

**Figure 1.**
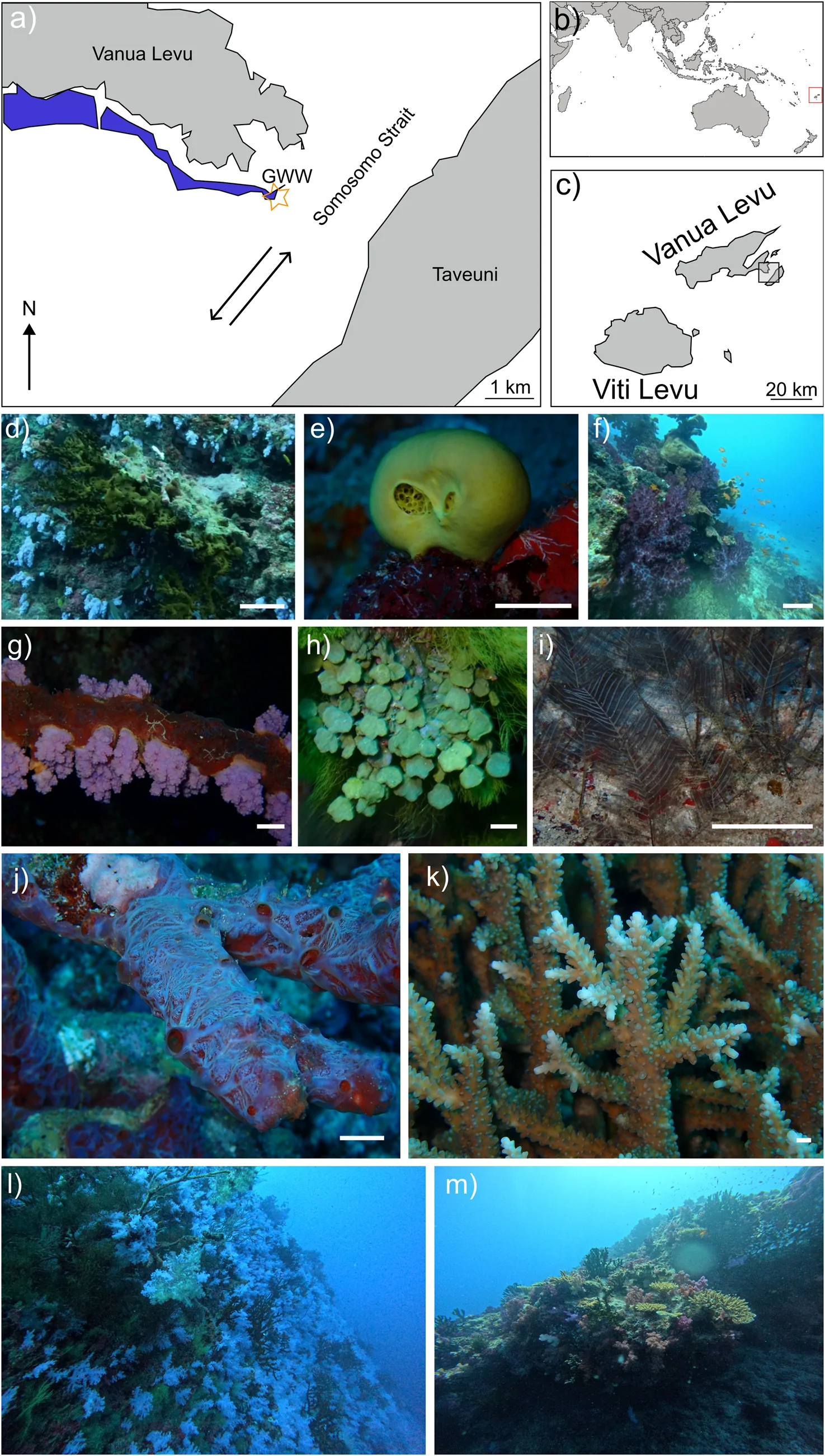
Rainbow Reef localities and morphospecies and groups of morphospecies. (a) Location of Rainbow Reef study sites in the Somosomo Strait, Fiji (approximate coordinates: (latitude: −16.795, longitude: 179.906); (b) Fiji's global location; (c) Rainbow Reef location relative to Viti Levu and Vanua Levu islands, Fiji. Arrows represent prevailing current directions; blue area represents barrier reef. Abundant reef morphospecies and groups of morphospecies: (d) *Tubastraea*, (e) lemon sponge, (f) *Dendronephthya* on the UFR, (g) White Nephtheidae, (h) *Halimeda*, (i) hydroid colonies, (j) encrusting sponge, (k) example of scleractinian (*Acropora*. sp.) Scale bars for (e,g–k) approximately 1 cm; scale bars of (d) and (f) approximately 10 cm. Example reefs: (l) the GWW view from approximately 17 m, and (m) the hard and soft coral dominated UFR at the top of the GWW at approximately 12 m.

Owing to the high degree of cryptic diversity present in soft corals, we assigned each organism to a ‘morphospecies’. Each morphospecies is approximated to a taxonomic group at genus level where possible (supplementary material, table S1). Across the three sites, we identified six abundant benthic morphospecies: (i) white Nephtheidae soft corals, (ii) colonial hydroids (possibly *Myriopathes*), (iii) lemon sponges (*Leucetta* cf. *chagosensis*), (iv) colourful *Dendronephthya* soft corals, (v) *Halimeda* algae, and (vi) *Tubastraea* (an azooxanthellate stony hexacoral). Further morphospecies found within the study sites but not in sufficient abundance (*n* < 100) for the analyses used here included: (i) sponges: *Stylissa*, *Rhabdastrella*, *Neopetrosia*, encrusting sponges and the endolithic sponge *Siphonodictyon*; (ii) other soft corals: *Sarcophyton*, *Cladiella*, *Klyxum* and *Chironephthya*; (iii) whip gorgonians; (iv) sea fans (possibly *Annela*, *Melithaea*, *Subergorgia* and *Acanthogorgia*); (v) Stylasterids; (vi) tunicates (*Polyandrocarpa*, *Didemnum*); (vii) holothurians (*Pearsonothuria*); (viii) crinoids (*Anneissia*); (ix) molluscs including mussels (*Pedum*, *Tridacna*), sea slugs and nudibranchs (*Phyllidia*, *Chromodoris lochi*, *Pteraeolidia semperi*); and (x) scleractinian corals (e.g. *Acropora*, *Pocillopora*, *Porites*, *Favites*).

### 2.2. Data collection

We used structure-from-motion photogrammetry to construct maps of each Rainbow Reef site following Stephenson *et al.* [46]. Video footage was collected in a lawnmower pattern and then split into frames at 1 frame s^−1^ using FFmpeg [47]; blurry frames were removed. For each site, frames were aligned to form three-dimensional models in Agisoft Metashape v2.1.1 with laser dots and objects of a known size used as scales. A two-dimensional ‘orthomosaic’ photo map was generated from each three-dimensional model and organisms were outlined and identified from each map with the morphospecies and location recorded. We used additional video and photographs to confirm morphospecies identification. Owing to the encrusting or sprawling body plans found in some taxa (e.g. encrusting sponges, some algae), individuals were identified from orthomosaics by the boundaries of those organisms, and each of these colonies were treated as a single unit. The marked-up maps were extracted using a custom script (available at https://github.com/nis38/NPS_dex, adapted from [48]) in R v4.2.2 [49] to produce spatial maps of morphospecies at each site.

### 2.3. Bayesian network inference

We performed BNI in software banjo (BNI with Java objects) v2.2.0 (for details, see [30,50]). We subset our spatial maps for BNI into 2 m^2^ quadrats (cf. [31]), and converted counts into density to account for the uneven edges of the mapped areas (supplementary material, table S2). Two square metres was chosen as this size balanced the expected spatial scale of ecological interactions, while also maximizing the number of quadrats used for BNI analyses. While we acknowledge that areal coverage is often used as a metric to quantify coral prevalence on reefs, the soft corals we focused on in this study change their body size dependent on flow conditions [27], and thus density of colonies provides a more comparable metric. Each quadrat was assigned a depth corresponding to either the UFR (12 m) or the depth bins identified in [46]: shallow (17–23.5 m), intermediate (23.5–28 m) and deep (28–33.5 m). Depth bin definitions were dictated by variation in GWW topography; the intermediate depth bin contains four shallow caves (fig. 2 in [46]), which are areas that interact with fluid flow (and thus food and light availability), whereas these caves were absent from shallow and deep bins.

The BNI algorithm required each variable be discretized, and used uniform priors to maximize statistical power and mask noise [30,51]. Morphospecies were discretized depending on the zero-weighting in the dataset following approaches for BNI applications to ecological datasets in previous studies (supplementary material, table S3):

— If greater than 70% of quadrats contained 0s for a given morphospecies, the morphospecies was re-grouped into an ecologically similar group of morphospecies (cf. [29–31,52,53]). If there was no ecologically similar morphospecies for re-grouping, then the morphospecies were considered too rare for BNI and SPPA.
— If less than 70% and greater than 40% of quadrats contained 0s for a given morphospecies and there was no other sensible re-grouping, the morphospecies was assigned presence–absence.
— If less than 40% of quadrats contained 0s for a given morphospecies, the morphospecies was assigned either a zero, low or high count, with low and high counts split by the median density across all quadrats after removing 0s.

The grouping of morphospecies into groups yielded two nodes in addition to the six high-abundance morphospecies: (i) a scleractinian group and (ii) an encrusting sponge group (supplementary material, table S1). While the scleractinian group necessarily included taxa which have disparate ecological roles [54,55], this allowed us to test whether there was any association between the density of scleractinians and any other variable within the BN, such as broad environmental preferences [56] or strong competitive effects [57] shared across the group. However, we advise caution in attempting to discern ecological associations between any group and all scleractinians from our results given plausible complexity of ecological interactions within the scleractinian genera that comprise this group.

BNs cannot normally be analytically found, so a search algorithm was required to find the best-fit BN which used Bayesian information criterion to find the best performing network (cf. [30,52]). A greedy search (maximizing nodes) with hill climbing was implemented (cf. [31]). To minimize outlier bias, we conducted 10 searches, where each search used a dataset of 1000 samples with a 95% bootstrap (with replacement; cf. [30]). Edge frequencies were bimodally distributed and used to define high and low probability edges. We constructed an averaged network by taking the mean edge frequency from the best networks from 10 searches and only the high-frequency edges (greater than 0.6) contributed to the final averaged network, with their IS calculated as a mean of the constituent edge IS [32]. For each edge, the directionality was the direction which occurred more often in BNs (greater than 50%) when the edge occurred.

### 2.4. Spatial point process analysis

To discern possible underlying processes that could underpin the edges recovered in BNI, we performed bivariate SPPA for taxa with sufficient sample sizes (*n* > 100 each) [58]. The pair correlation function (PCF) was used to test model fits between the observed point pattern of each edge against known spatial patterns [45]. PCFs represent how the density of points (e.g. corals) at a given distance *r* from a given point change as a function of distance from that point [45]. While inferring ecological processes from patterns is difficult because of the possible superposition of multiple processes (i.e. the overlaying of intra- and interspecific interactions or variable environmental preferences) and equifinality (where different processes generate similar spatial patterns) [43,59–61], the application of complimentary statistical techniques can disentangle processes underpinning different ecological patterns, particularly if those patterns occur across differing spatial scales [43,60]. We performed spatial analysis in Programita (v. Novembre 2018) [62].

For the spatial analysis, since the only edges that met the sample size requirements were positive, we tested random and aggregation models only, namely : (i) homogenous Poisson, or complete spatial randomness (CSR), a suitable null model for CSR indicating no interactions or associations over the spatial scales tested [43,63]; (ii) a heterogeneous Poisson (HP) process, modelling aggregation owing to habitat associations; (iii) a linked-Thomas cluster (LTC) process, modelling aggregation owing to facilitation [31,64–66]; (iv) a shared-parents cluster (SPC) process, modelling bivariate aggregation around a shared point owing to an unmeasured shared facilitative third species or mutually beneficial resource; (v) an inhomogeneous LTC (ILTC) process, modelling facilitation in areas of high habitat suitability; and (vi) an inhomogeneous SPC (ISPC) cluster process, modelling bivariate aggregation around a shared point owing to an unmeasured shared facilitative third species or mutually beneficial resource in areas of high habitat suitability.

Identifying a suitable null model for CSR, indicating no ecological association between species, is non-trivial because a bivariate pattern is composed of two univariate component patterns which might have been created by a potentially different set of processes (e.g. intraspecific associations, competition). Dispersal limitation is known to be a dominant process for populations on the GWW [46], and so a null model was required that could account for these univariate patterns. A standard null model for this approach is a toroidal shift [43,67], where one univariate pattern (pattern 2) is shifted a whole random vector against another pattern (pattern 1); however, our sample areas were irregular in shape, and so inappropriate for a toroidal shift approach [43,67]. Instead, in addition to a CSR process with no modification, we fitted a parametric point process—in this instance, a Thomas process—to one of the component patterns (pattern 2) and used the realizations of the fitted point process as a null pattern for independence in Programita (v. Novembre 2018) [62].

For HP, ILTC and ISPC analysis, habitat heterogeneity was approximated in one of three ways:

— By smoothing the density map of a morphospecies indicating a shared response to an unmeasured habitat variable (HP_latent_, ILTC_latent_, ISPC_latent_) [68].
— By using the background topography (i.e. substrate height) from a digital elevation model of the GWW, extracted from photogrammetry (HP_topog_, ILTC_topog_, ISPC_topog_; cf. [46]).
— By using a depth gradient (HP_depth_, ILTC_depth_, ISPC_depth_).

Note that we could not fit multiple approximations of habitat heterogeneity in a single model because this is not possible in Programita [62] and, while this technique is possible in the R package *spatstat* [69], *spatstat* does not yet have the capability to test bivariate clustering processes (e.g. SPCs). For HP_latent_, we produced density maps of each morphospecies using an Epanechnikov kernel with a bandwidth of 0.5 m, a value that consistently provided a balance between the locations of the organisms and the variation generated by them (cf. [31]). To test whether a bivariate PCF was best fitted to a given null model PCF, 999 Monte Carlo simulations were run for each process, and the simulation envelopes taken to be between the 5% highest and lowest values (cf. [43]). CSR and HP models were fitted using maximum likelihood methods and LTC, SPC, ISPC and ILTC models were fitted using minimum contrast methods [70,71].

A goodness-of-fit test was then used to quantitatively assess differences between the observed pattern PCFs and simulated PCFs. Goodness-of-fit tests provide a hypothesis test, with the null hypothesis being that the measured process (here, PCF for each pair of morphospecies' point patterns) departs from the simulation envelope over a specified distance interval [43,72]. Here, we used Diggle's goodness-of-fit test (*p_d_*), with high *p_d_* (i.e. closer to 1) interpreted to be a good model fit [72], alongside visual inspection of the morphospecies' PCF plot [43].

### 2.5. Rare morphospecies and diversity analysis

In most ecosystems, the majority of species are rare [73,74] and the identity of rare species may vary with depth or the dominant group (e.g. scleractinian versus soft corals). For the BNI and SPPA, rare species could not be included in our analyses [30] so, to investigate the possible effects of rare taxa, we removed all common morphospecies for each quadrat to create a dataset of rare morphospecies. We used a linear discriminants analysis (LDA) with depth as a predictor variable and density of each rare morphospecies as response variables. An LDA is a supervised ordination method whereby clustering is dictated by the predictor variable [75]. The accuracy of the LDA was tested using a training–testing split of 60 : 40, whereby 60% of the data was used to construct the LDA, and the results of the LDA were used to predict the remaining 40% of the data.

In order to assess the effects of the soft corals and depth on the diversity of each site, Simpson's index [76], Shannon evenness [77,78] and Fisher's alpha parameter [79] were calculated for each quadrat. Each metric was used as a response variable in separate linear mixed models using the R package *lme4* [80] with depth bin and density of white Nephtheidae as fixed response variables and site (UFR, GWW and ARW) as a random variable in each model.

## 3. Results

White Nephtheidae were the dominant morphospecies on the intermediate and deep depth bins of GWW (87% relative abundance) and in the intermediate depth bin of the ARW (80% relative abundance). Mixed scleractinian and soft corals (including the white Nephtheidae) were present in the shallow GWW (6% scleractinian, 66% soft coral) and the shallow ARW (30% scleractinian, 9% soft coral). The UFR was evenly split between scleractinians (47%) and soft corals (48%, mostly colourful *Dendronephthya*; table 1).

**Table 1.** Sample sizes for all morphospecies that appeared frequently enough to be included in Bayesian network (BN) analysis on the rainbow reef; UFR = upper forereef, GWW = Great White Wall, ARW = adjacent reef wall.

		GWW			ARW		
morphospecies	UFR	shallow	intermediate	deep	shallow	deep	total
white Nephtheidae	0	1417	9355	10 943	6	2818	24 539
*Dendronephthya*	348	153	86	23	28	55	693
*Tubastraea*	32	128	94	73	9	16	352
encrusting sponges	2	99	62	38	7	6	214
lemon sponges	2	260	93	96	156	130	737
*Halimeda* algae	0	155	462	329	0	0	946
hydroids	6	159	316	584	55	128	1248
Scleractinians	342	22	13	11	98	24	510

### 3.1. Bayesian network inference

Nine nodes (eight morphospecies/groups of morphospecies and depth) were included in the best BN with a linkage density of 0.78. Depth was the dominant node directly associated with the density of the white Nephtheidae and hydroids positively, and *Dendronephthya* negatively. Depth was indirectly associated with all other nodes apart from lemon sponges, which had no edges. The white Nephtheidae were positively associated with *Halimeda* algae and negatively associated with scleractinians. *Dendronephthya* were positively associated with *Tubastraea*, which in turn were positively associated with encrusting sponges (figure 2).

**Figure 2.**
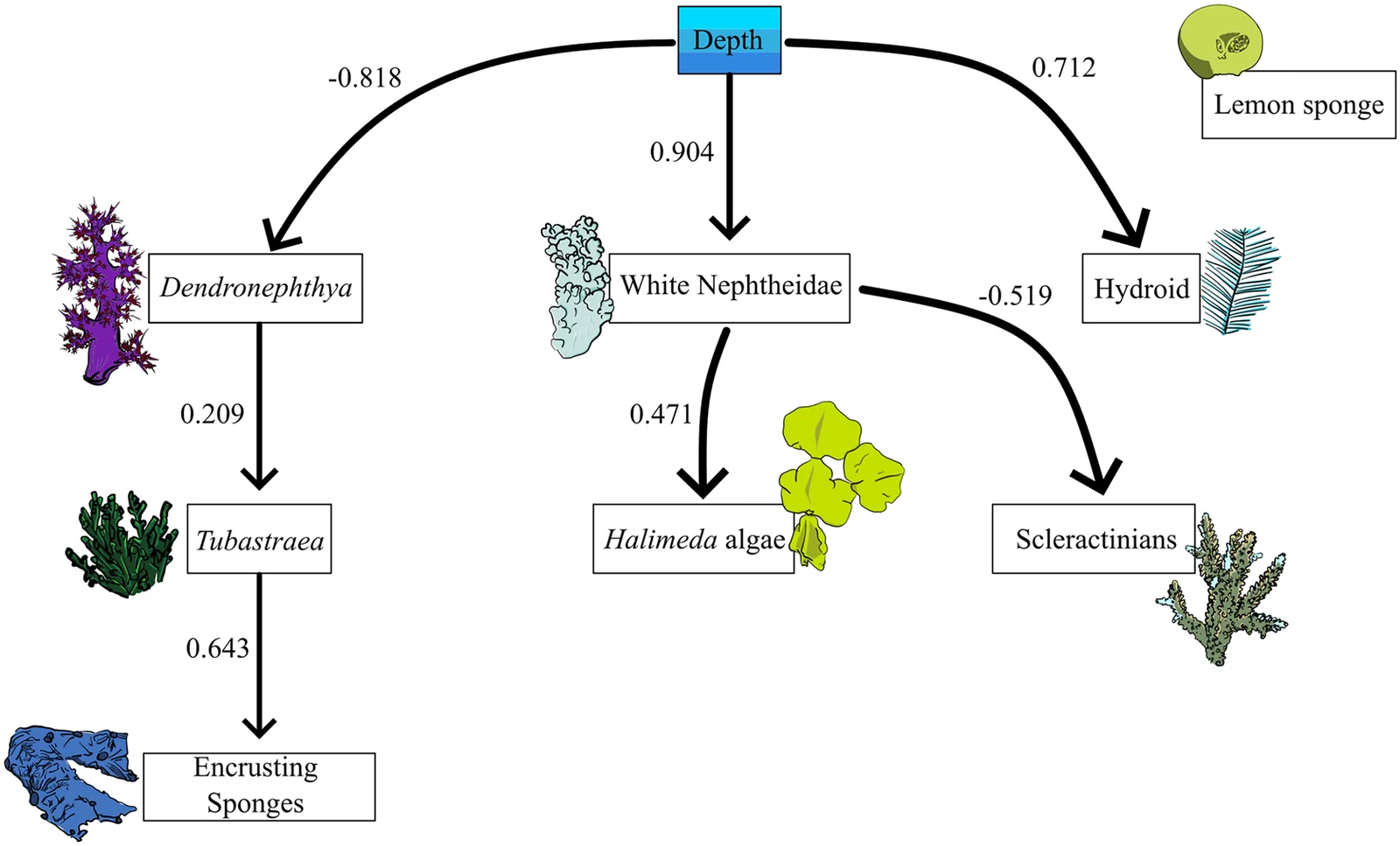
Bayesian network (BN) of associations on the Rainbow Reef. Thickness of edges reflects occurrence of edges (included in greater than 60% of networks), arrows denote directional effects, numbers next to edges indicate the influence score (IS) (strength and sign of interaction).

### 3.2. Spatial point process analysis

There were four pairs of taxa that were sufficiently abundant for SPPA: *Dendronephthya* and *Tubastraea* in the shallow GWW, and white Nephtheidae and *Halimeda* algae at the shallow, intermediate and deep depth bin of the GWW. To avoid secondary co-correlations, spatial associations were only tested using SPPA in the direction that corresponded to the association identified by BNI (*Dendronephthya* on *Tubastraea*, white Nephtheidae on *Halimeda* algae).

The best-fit model for the *Dendronephthya–Tubastraea* relationship was spatial independence once univariate clustering of *Tubastraea* had been accounted for (*p_d_* = 0.587, figure 3; supplementary material, table S4). The best-fit model for the white Nephtheidae–*Halimeda* algae relationship in the GWW shallow depth bin was an ILTC*_d_* model (*p_d_* = 0.423) indicating a good fit to a facilitative interaction where the white Nephtheidae facilitate *Halimeda* algae associated with increasing depth. We found the best-fit model for the white Nephtheidae–*Halimeda* algae relationship in the GWW intermediate depth bin was spatial independence parameterized for the clustering of the *Halimeda* algae (*p_d_* = 0.480; supplementary material, table S4). We found a good fit (*p_d_* = 0.474) for an ILTC_latent_ (habitat-mediated facilitation) model for the white Nephtheidae–*Halimeda* algae relationship for the GWW deep depth bin, indicating a good fit to a facilitative interaction where the white Nephtheidae facilitate *Halimeda* in areas where there is high habitat suitability for *Halimeda*.

**Figure 3.**
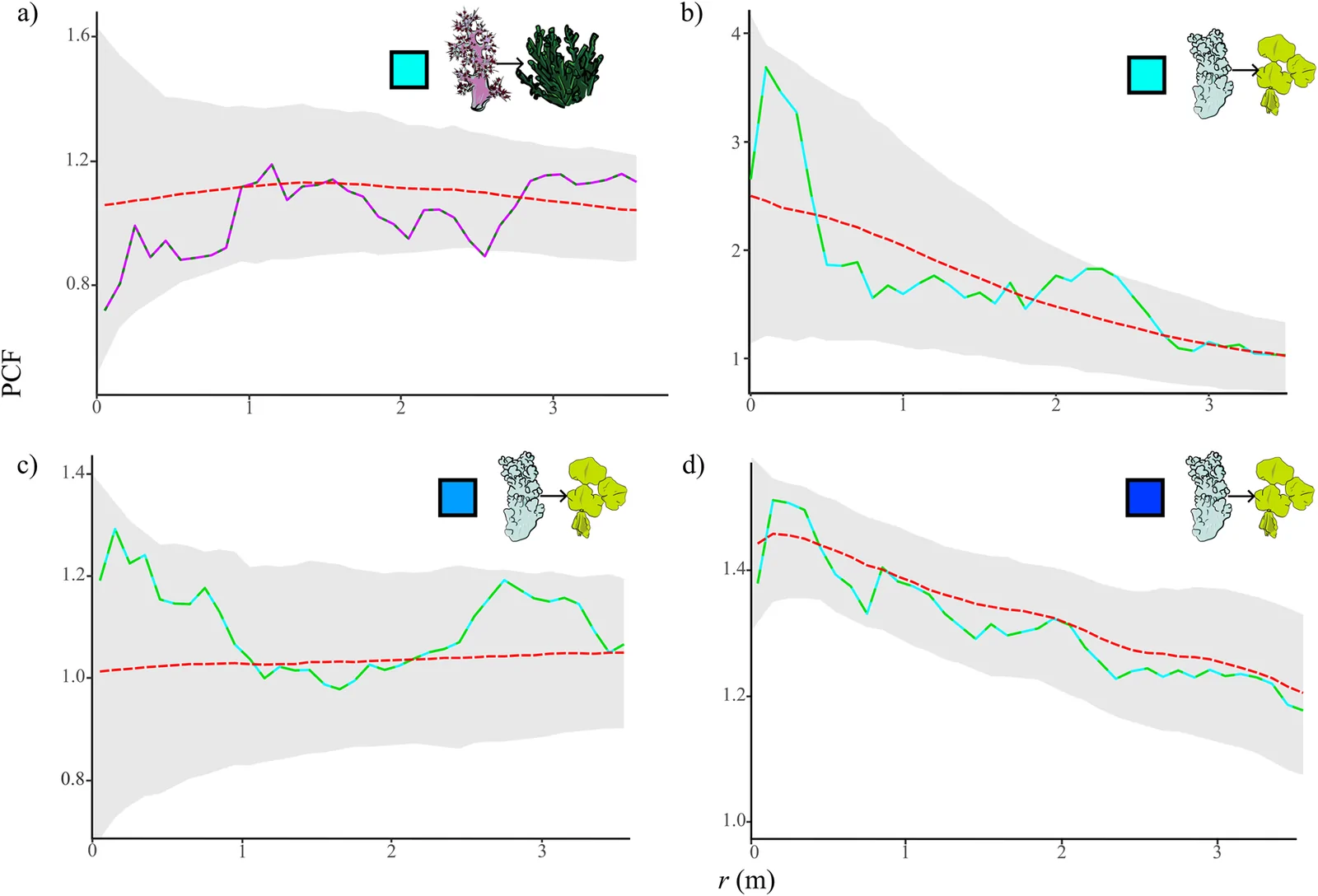
PCF (ordinate) plots of bivariate SPPA for abundant morphospecies identified to directly interact via BNI (figure 2) at different depths of the main GWW. (a) *Dendronephthya* effect on *Tubastraea* compared to a CSR simulation envelope accounting for *Dendronephthya* clustering; (b) white Nephtheidae effect on *Halimeda* algae compared to an ILTC (depth-mediated facilitation) simulation envelope; (c) white Nephtheidae effect on *Halimeda* algae compared to a CSR simulation envelope accounting for white Nephtheidae clustering; (d) white Nephtheidae effect on *Halimeda* algae compared to an ILTC (habitat-mediated facilitation) simulation envelope. Blue/green (white Nephtheidae-*Halimeda*) and pink/green (*Dendronephthya-Tubastraea*) lines indicate bivariate PCF for each morphospecies pair. Shades of blue indicate depth bin (light is shallow, intermediate is intermediate, dark is deep). Dashed red lines indicate fitted model (a,c: CSR; b: ILTC_depth_; d: ILTC_latent_).

### 3.3. Rare morphospecies and diversity analysis

To infer the effect of rarer taxa, our LDA, including only rare taxa, established three overlapping clusters representing the shallow, intermediate and deep depth bins across all sites (figure 4a). Note that the UFR was removed owing to low sample size after implementation of the testing–training split. The LDA was a good predictor of true depth (64% accuracy). Visual inspection suggested that rare morphospecies diversity was determined by the density of white Nephtheidae (figure 4d, red box and arrow).

**Figure 4.**
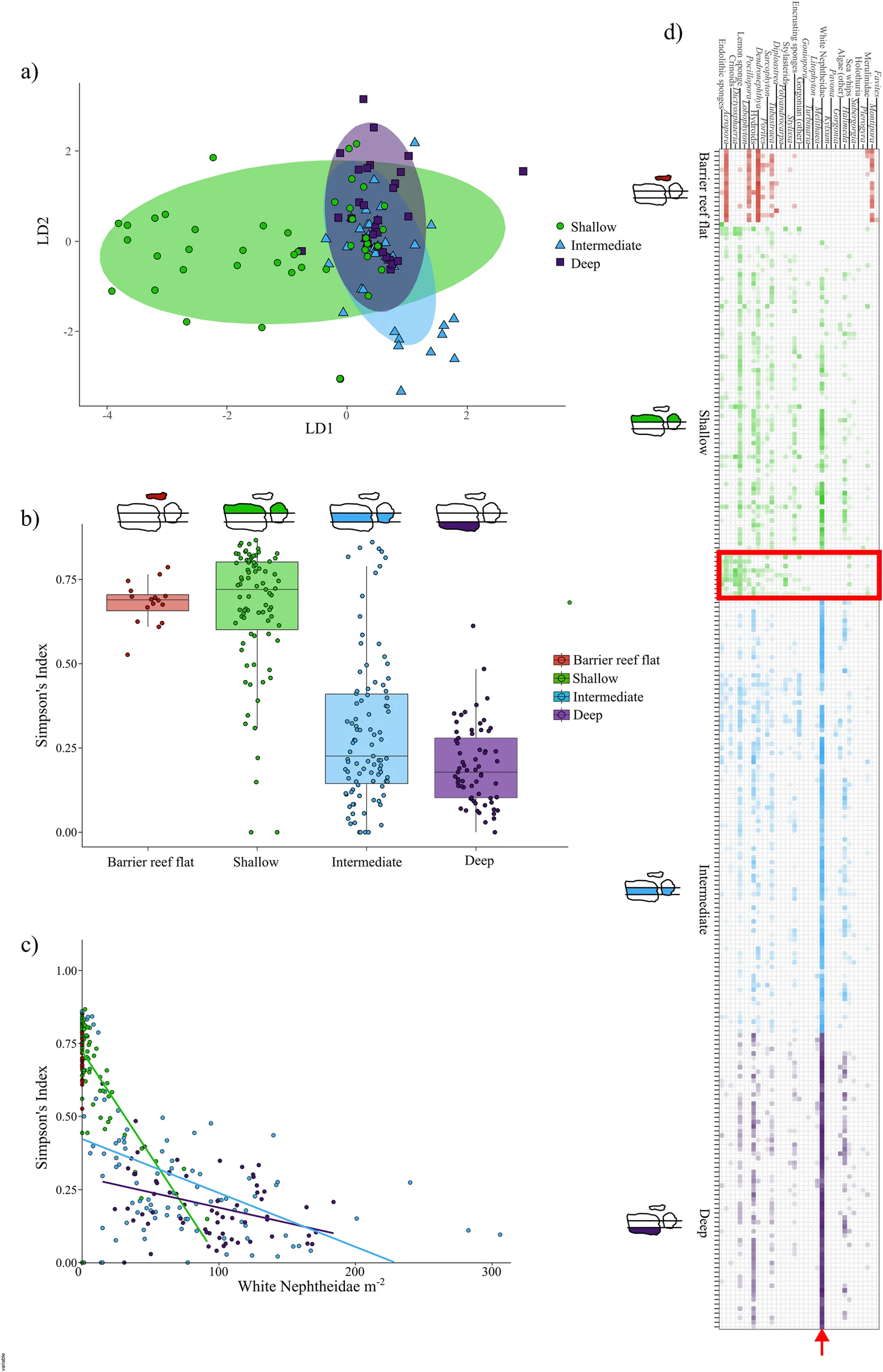
Composition and diversity analysis on the Great White Wall (GWW) and adjacent reefs. On all plots, red = barrier top, green = shallow depth bin, blue = intermediate depth bin, purple = deep depth bin. (a) An LDA of the rare taxa, symbols indicate depth bin (circle = shallow, triangle = intermediate, square = deep) and ellipses indicate range occupied by each depth bin; (b) differences in Simpson's diversity index between different depth bins, with points overlayed with a jitter; schematics indicate depth bin; (c) association between Simpson's diversity index with the density of white Nephtheidae, with colours indicating relationships within depth bins; (d) matrix plot of morphospecies densities in each quadrat on the GWW and adjacent reefs, the opacity of each cell indicates log density (more opaque = more dense); schematics indicate depth bin. Red boxes indicate species rich patch with absence of white Nephtheidae soft corals (row indicated by red arrow).

When testing the drivers of diversity for all morphospecies, we found that Simpson, Shannon and (log transformed) Fisher alpha diversity indices were all negatively associated with the increasing depth bin (mean effects on Simpson's index: shallow: −0.002; intermediate: −0.253; deep: −0.309; figure 4b), but 95% confidence intervals crossed zero for all depth bins for all diversity indices apart from the deep depth bin, which had a significant negative effect on Shannon's index (supplementary material, table S5). We also found that there was an overall negative association between Simpson, Shannon and (log transformed) Fisher diversity indices and the density of white Nephtheidae (mean effect Simpson's index −0.004), and that 95% confidence intervals did not cross zero, indicating that the white Nephtheidae may be the main driver of rare taxa diversity (figure 4c; supplementary material, table S5).

## 4. Discussion

In this study, we investigated likely ecological interactions between morphospecies across three sites on the Rainbow Reef, a soft coral-dominated reef in Fiji, using BNI and SPPA. We found a combined effect of depth and soft coral density was associated with ecological and diversity dynamics. Depth was the most influential node in the BN, affecting all morphospecies except for lemon sponges, and this pattern is reflected in previous work showing that depth influenced the population ecology of filter feeders and algae on the GWW [46]. In terms of edges between biotic nodes, *Tubastraea* had a positive association with encrusting sponges in the BN, but sample size precluded any subsequent spatial analysis. It could be that *Tubastraea* acts as suitable habitat for encrusting sponges, as they are known to grow over branching scleractinians [81]. *Dendronephthya* had a positive association with the density of *Tubastraea*, but the best-fit model from SPPA was random (CSR) across the spatial scales tested (0–3.5 m; figure 3a; supplementary material, table S4). We therefore consider the positive relationship between *Dendronephthya* and *Tubastraea* recovered from BNI to potentially reflect large-scale habitat preferences since both are azooxanthellate. The lemon sponges had no strong association with any variables in the network. Previous work found that lemon sponge density was relatively consistent across all GWW depth bins [46], and there was a minor effect of depth on the size and density of lemon sponge reproductive clusters (identified in [46]) compared to *Halimeda* algae, hydroids and white Nephtheidae, so lemon sponges may have broader biotic and abiotic tolerances than could be tested here. Scleractinians and white Nephtheidae shared the only negative edge between biotic variables in the network, which may reflect segregation arising from competition or distinct habitat preferences, but these effects could not be tested by SPPA since the groups never spatially overlapped in sufficient abundance (*n* = 100 each). Competition between soft corals and scleractinians has previously been described via allelopathy, which precludes settling of scleractinians and could be a cause of the segregation (via competitive exclusion) of the two morphogroups between the GWW sites [80], particularly where environmental factors are more ideal for soft corals. However, large-scale habitat preferences (e.g. variation in light, substrate, flow regime) between walls and forereefs are more likely drivers given the differences in niche use between zooxanthellate scleractinians and azooxanthellate Nephtheidae soft corals.

While previous work has highlighted competition between algae and scleractinian corals [82], we found no algae–scleractinian coral edges in our network. Note though that, owing to limits in sample size, we did not separate the scleractinian node into genera with known ecological distinctions (e.g. *Acropora* and *Porites*) [54,55], and so we are unable to comment on the complex and diverse interactions in the scleractinian corals. No association between these nodes may therefore be unsurprising given the lack of taxonomic resolution in the scleractinian node, especially given that coral–algae competition is often species-specific [83,84]. The lack of direct association could also be a reflection of relatively low impact of human disturbance on the Rainbow Reef, which has been shown to modulate coral–algae competition, for example, via overharvesting of grazers [85,86]. We instead found a positive association between white Nephtheidae soft corals and *Halimeda*, and a negative effect of white Nephtheidae on scleractinians, resulting in an indirect negative association between scleractinians and algae mediated by soft corals, but we cannot identify the core interactions that drive these associations.

Subsequent SPPA on the white Nephtheidae–*Halimeda* algae relationships showed that the best-fit model for the white Nephtheidae–algae interaction indicated facilitation and habitat associations (ILTC process) in the shallow and deep depth bins. While it is not possible to disentangle the cause of this facilitation using our data (see §1), it could be that the algae grow in areas where soft corals have suppressed grazing activity via production of toxic compounds [87,88] or protection mutualisms imparted by invertebrates such as pistol shrimp [89], so the positive edge and best-fit spatial models could reflect an indirect interaction (figure 5). In the intermediate depth bin, the best-fit model indicated no association between the white Nephtheidae and *Halimeda*. The positive association between these taxa in the BN may reflect large-scale shared habitat preferences. Given the facilitative relationship found in the deep and shallow depth bins, the poor fit to facilitative spatial models in the intermediate bin might be due to the superposition of changing ecological dynamics [43] with depth or habitat variability (as shown on the GWW in [46]). Interestingly, *Halimeda* were not found on the ARW where white Nephtheidae were present but in lower densities (although other morphospecies of algae were present in low densities). The difference between the GWW and the ARW could reflect a range of ecological interactions such as: (i) the facilitation by white Nephtheidae is not strong enough on the ARW owing to lower density, (ii) a dispersal limitation of the *Halimeda* algae, (iii) the habitat for algae was of a poorer quality, and/or (iv) the relationship is non-monotonic (e.g. thresholded density-dependence [90,91]). Given that *Halimeda* algae and some soft corals can replace scleractinians on tropical coral reefs after bleaching and prevent recovery to a scleractinian-dominated state [6,15,92], a positive association between *Halimeda* algae and soft corals might be a potential impediment to post-disturbance recovery, particularly if soft corals can depress grazing pressure.

**Figure 5.**
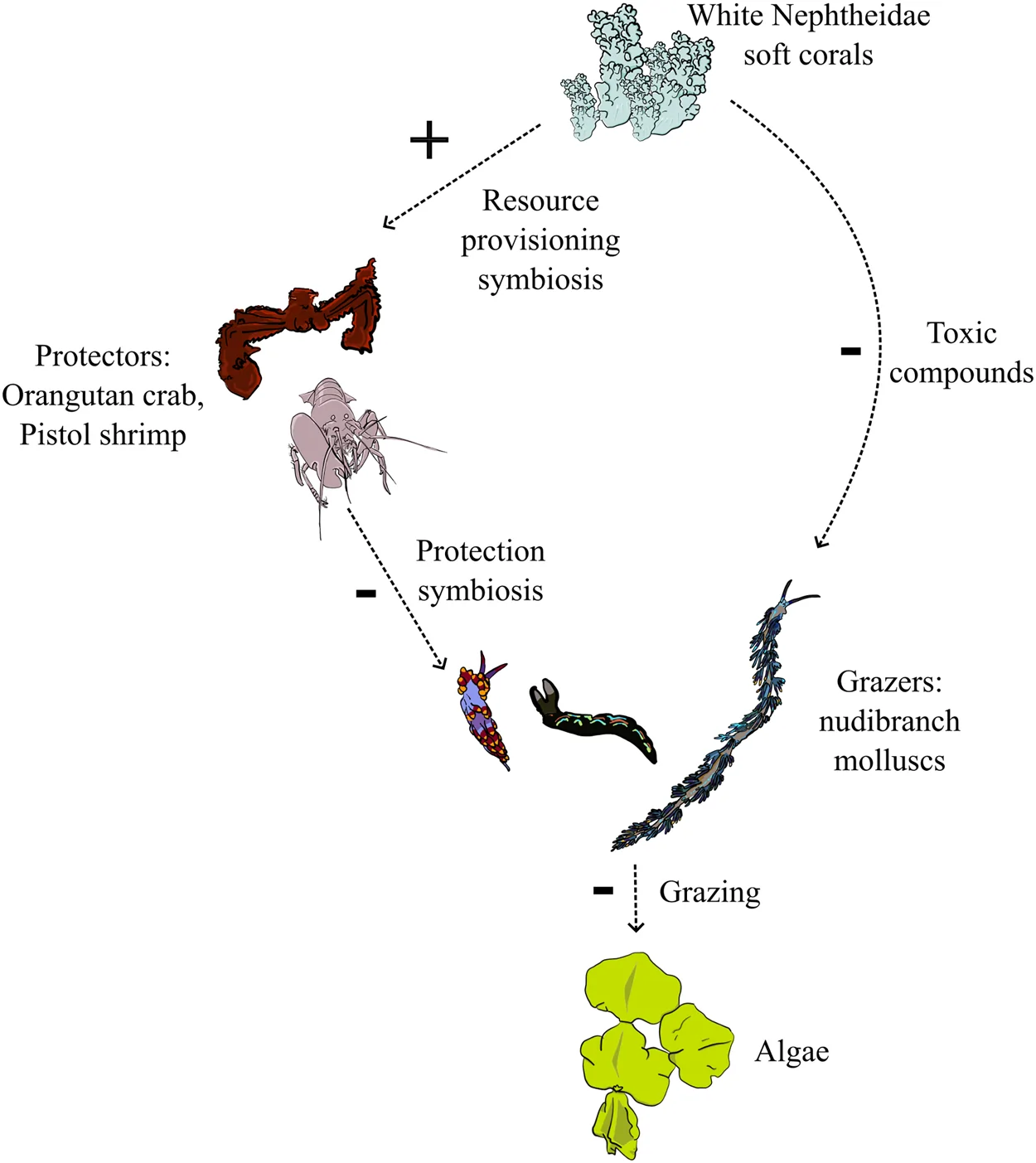
Putative network schematic of plausible facilitation between white Nephtheidae soft corals and *Halimeda* algae (figures 2 and 3d). White Nephtheidae deploy toxic compounds or facilitate protective mutualists to deter grazers, depressing the local impact of grazing. The release of grazing pressure generates favourable conditions of algal growth, so white Nephtheidae and algae are more commonly found together. We observed all organisms figured here on or near the GWW, apart from the pistol shrimp, which has been found to be associated with Nephtheidae soft corals at other sites [89].

Although the white Nephtheidae soft corals have a positive spatial relationship with algae in the shallow and deep depth bins, there is an overall negative association between the density of white Nephtheidae on the diversity of any 2 m^2^ quadrat in which they are found, indicating that these soft corals may suppress benthic reef diversity on the GWW. While previous work suggested that Nephtheidae soft corals may elevate benthic diversity through temporal niche partitioning (whereby intervals of feeding could yield periods where other filter-feeding benthos can make use of resources in low-current conditions [46]), compared to the UFR, the benthos was significantly less diverse in quadrats where white Nephtheidae density was high (figure 4c). This relationship could, in part, be due to the biodiversity generated by the three-dimensional microhabitat engineered by scleractinians on the UFR [24]. The strong abiotic differences between these habitats (flow regime, nutrient and light availability, substrate topography) are also probable key drivers of benthic biodiversity, but how these factors interact with the density of soft corals is still not known. While Nephtheidae soft corals also possess large three-dimensional bodies, the structural habitat they generate is ephemeral owing to changes in their morphology during periods of low current [27]. This transient structural complexity could reduce nursery, sheltering or wave buffering functions compared to scleractinian-dominated reefs. It is possible that a reduction in grazers or corallivores suppresses the biodiversity benefits generated through intermediate disturbance since these soft corals are resilient to removal by wave action, fast currents or physical removal by reef predators while hunting (as seen with scleractinians and reef sharks [93,94]). Previous work has similarly reported that fish diversity is lower in soft coral-dominated reefs compared to scleractinian reefs [20], although see [95]. We found that the composition of rare taxa varied with depth, which could be driven by white Nephtheidae density. We also found that depth was a key contributor to this loss of diversity, which itself is correlated within increasing density of white Nephtheidae, and it is challenging to disentangle the causative element.

Scleractinians and Nephtheidae soft corals are challenging to compare because of structural differences in their body plans; scleractinians can be large, and are often best modelled by aerial coverage (e.g. [84]), rather than by points. Nephtheidae soft corals undergo dramatic size changes [27], and so points or abundance better represents their ecological dominance. While our BNs account for this by normalizing data within a node (here, morphospecies) via our discretization process, how we measure the dominance of these groups probably affects our diversity analyses, where count data were preferred to body size as a method for comparing scleractinian and Nephtheidae dominance. Similar issues arise with morphospecies where discerning individuals from colonies is challenging, particularly from orthomosaics (e.g. encrusting sponges).

Our work highlights the joint role of environment and potential species interactions in determining site species composition and diversity, in addition to the role of dispersal limitation [46,96]. We show that soft coral-dominated quadrats on the Rainbow Reef are composed of a different, and less diverse, assemblage of benthic taxa, than nearby scleractinian-dominated quadrats, which is concerning given that future climate change may promote soft coral abundance on coral reefs [21]. Depth was a key environmental variable in our network, highlighting the transition from photosymbiont-hosting scleractinians to aphotosynthetic filter-feeder communities. We also show that the facilitative relationship between white Nephtheidae and algae, which could be due to reducing grazing pressure, occurs most strongly where the white Nephtheidae dominate. Finally, if our results from the GWW apply more generally, this suggests that reef walls, particular those dominated by Nephtheidae soft corals, exhibit reduced benthic diversity—potentially because these soft coral reef systems are underpinned by different ecological dynamics.

## Data Availability

The R code to reproduce this work is available at https://github.com/nis38/GWW_BNI and the data are available at ( doi:10.6084/m9.figshare.28650257).
